# Topologically protected plasmonic phases in randomized aperture gratings

**DOI:** 10.1038/s41598-023-28022-3

**Published:** 2023-01-18

**Authors:** Maayan Fox, Yuri Gorodetski

**Affiliations:** 1 Electrical and Electronics Engineering Department, 407000 Ariel, Israel; 2 Ariel Photonics Center, 407000 Ariel, Israel; 3 Mechanical Engineering and Mechatronics Department, 407000 Ariel, Israel

**Keywords:** Optical physics, Metamaterials, Photonic crystals

## Abstract

We experimentally show the excitation of surface plasmons by topologically protected diffraction from gratings with randomized periodicity. The structures are designed such that the plasmonic excitation is conditioned by the proper combination of the geometric and the dynamic phases. Accordingly, it is possible to obtain a precise interaction of the incident light signal and a specific plasmonic directional mode in a polarization dependent manner.

## Introduction

**Figure 1:**
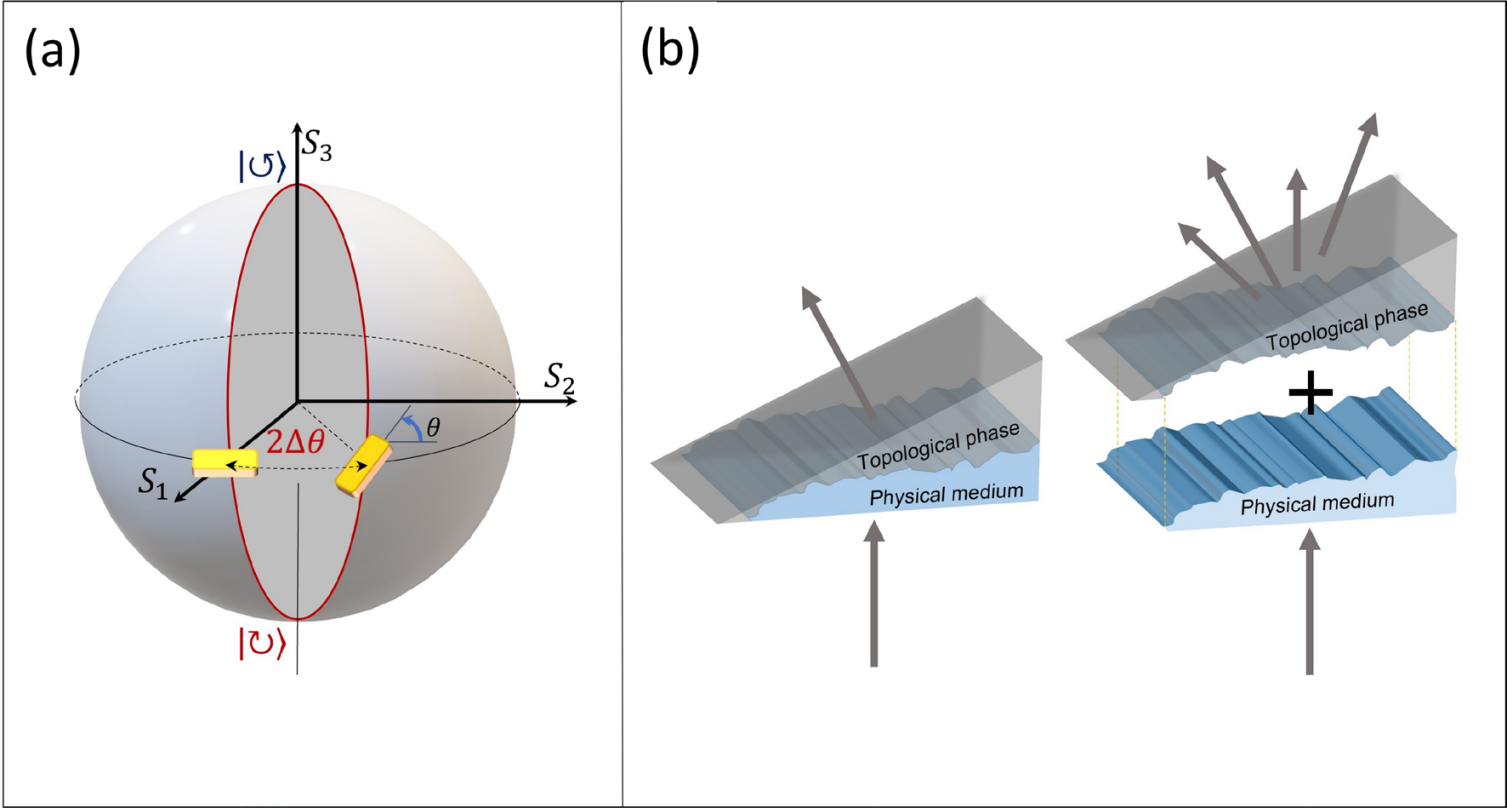
(**a**) Visualization of the TP on the Poincaré sphere due to rotating apertures. The angle of the anisotropy axis, $\theta$, corresponds to the longitudinal line of the sphere with the same angle. Subsequently, the solid angle subtended (in red) is then the topological phase accrued by coherent light passing through both apertures. (**b**) Graphic presentation of the combined effect of the plasmonic grating and the topological phase. The randomized physical medium and the topological phase apart scatter incident light in all angles, but once coupled the light is diffracted in a topologically protected direction.

Topological plasmonics is a relatively new and promising field of research due to the unique part that topology plays in phenomena such as polarization dependent plasmon scattering^1–3^, plasmonic topological insulators^4–7^, and more^8–10^. The Pancharatnam-Berry phase, or the topological phase (TP) has recently attracted a significant interest^11–13^, with notable implications for nano-optics^14,15^ and specifically plasmonics^16–18^. The polarization state-space of light is defined by the Poincaré sphere, taking on the non-trivial topology of genus zero. The TP can be accrued by driving the light state along a closed path on this sphere^19,20^. The area enclosed by this path (see Fig. 1a), which can be normalized to the solid angle subtending it in the case of a constant or slowly varying beam magnitude, is hence topologically protected.

Light propagating in free space can undergo a continuous space variant polarization modulation in order to achieve smoothly varying Berry phases^21^. Recently, it has been shown that TP can also be introduced in the process of surface plasmon (SP) wavefront excitation^1–3^. A grating comprising spatially rotating apertures was used to obtain two effects simultaneously—coupling incident light to SPs and imparting upon that light a geometric phase. As a result, the illumination of the structure by right-handed (or left-handed) circular polarization (RCP, LCP respectively) led to the unidirectional spin dependent excitation of the SP modes^22^. There the fundamental grating periodicity and its rotation rate were constant along the structure giving rise to a periodic diffraction pattern as could be expected from the Bloch–Floquet theorem. That resulted in the fact that both ordinary diffraction (OD) and topological diffraction (TD) orders were repeated periodically in *k*-space. Accordingly, an infinite number of the OD modes appeared in the far-field, each accompanied by a pair of the TD orders. 

Here we propose to consider a plasmonic grating with *no* defined local periodicity or unit-cell rotation rate. For this we randomize the dynamic phase (accrued by the plasmonic path between two adjacent apertures) and the TP (arising from the relative rotation between two adjacent apertures) while maintaining one condition—that their sum equal $2\pi$ everywhere on the sample. This structure is shown to support the excitation of unidirectional topologically protected plasmonic modes. Randomized optical structures in themselves have been a topic of some interest over the passing decades, having been implemented among other things in suppression of unwanted diffraction^23,24^, generation of specific intensity distributions at optical^25^ and x-ray^26^ frequencies, optical coding^27^, in one instance leveraging TP^28^, and optimization of solar cells^29^ and light emitting diodes^30^. Additionally, theoretical and computational models^31–33^ have been constructed for the understanding and prediction of such chaotic or randomized diffraction sources.

Our study clearly shows that the combination of the randomized grating periodicity compensated for by a corresponding topological phase yields one, and only one pair of *non-random, topologically diffracted plasmons*. Additionally, the remaining modes diffracted from the grating are uniformly scattered into the *k*-space, ergo leaving a uniform background. This de-coupling of topological and ordinary diffraction provides additional degrees of freedom and insight into topological photonics, potentially setting the stage for both new devices which wish to leverage purely topological states of light, and pioneering experimental concepts in analogous topological solid state physics.

## Results and discussion

Previous work^22^ has shown that the total momentum of a topological diffraction mode from a periodic grating with spatial rotation of the unit-cell can be described via the following equation:

1 $$\begin{aligned} k_t = k_{in} + nk_{g} + \sigma k_{\Omega } \end{aligned}$$

where $k_{in} = \frac{2\pi }{\lambda _0}\sin \alpha$ is the incident momentum of the beam (with $\lambda _0$ being the wavelength and $\alpha$ is the incidence angle) and $k_{g} = 2\pi /\Lambda$ is the fundamental momentum quanta imparted by the grating with a period of $\Lambda$. Here we suppose that both, the actual grating periodicity and the rotation of the unit-cell are along the same axis. The magnitude of the topological momentum $k_{\Omega } = 2\Omega$ is defined by the rotation rate of the apertures ($\Omega = d\theta / d\xi$, where $\theta$ is the local orientation of the unit-cell and $\xi$ is the coordinate along which the rotation is carried out) while the incident light spin number $\sigma$ takes the values of 1 for RCP and $-1$ for LCP state.

As suggested in the previous chapter, we now randomize the grating periodicity so that Eq. (1) can be only satisfied locally. For normal incidence ($\alpha = 0$) and $n = 1$ the local momentum delivered by the grating reads as, $k_t = \frac{2\pi }{\Lambda _i}+\sigma \frac{2\Delta \theta _i}{\Lambda _i}$ where the index *i* represents a local value. Now, by setting the coupling condition $k_t = k_{SP}$ (where $k_{SP} = k_{in}\sqrt{\frac{\varepsilon _m}{1+\varepsilon _m}}$, and $\varepsilon _m$ is the real part of the dielectric constant of gold) we obtain the requirement for the local grating period and rotation,

2 $$\begin{aligned} k_{SP}\cdot \Lambda _i -\sigma \cdot 2\Delta \theta _i =2\pi \end{aligned}$$

Here $\Delta \theta _i$ in radians denotes the relative rotation between the *i*th and $(i+1)$th apertures (see Fig. 3). It is now clear that Eq. (2) describes the condition for the constructive interference of plasmons at two adjacent apertures, taking into account the optical path (first term on the left side) and the TP contribution (second term on the left side) due to the relative rotation angle of $\Delta \theta _i$. We note that while $\Lambda _i$ and $\Delta \theta _i$ are varied along the grating the total grating momentum quanta at each period is constant which makes it possible to define the effective grating spacing as,

3 $$\begin{aligned} \Lambda _{eff}\triangleq \frac{\Lambda _i}{1+\sigma \cdot \frac{\Delta \theta _i}{\pi }} \end{aligned}$$

where $\Delta \theta _i$ takes values between $-\frac{\pi }{2}$ and $\frac{\pi }{2}$. This effective constant represents the distance in space between two topologically protected phase fronts, and is set here to match that of the surface plasmon. Interestingly, one can think about the proposed system as if the total desired phase of the beam is acquired by combining two contributions (topological and dynamic phases) while each one of them is randomized. This situation is graphically visualized in Fig. 1b as a fractured prism in two pieces representing the two aforementioned phase contributions. Illuminating each part separately will lead to the broad scattering of light, while combining them together brings about the correct phase front for the desired polarization.

**Figure 2:**
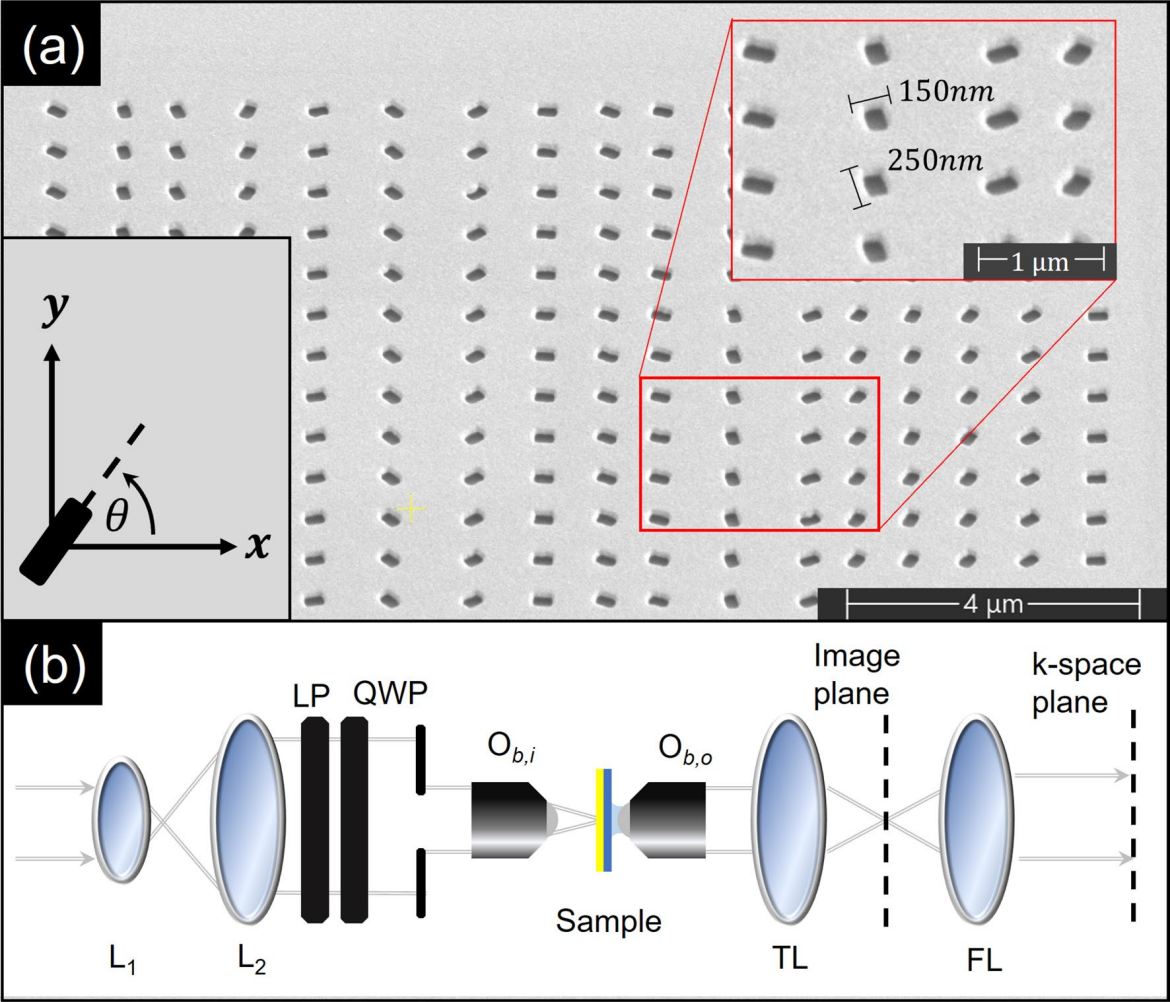
Randomized topological plasmonic structure. (**a**) SEM image of FIB milled apertures in gold. The inset schematically depicts characteristic aperture dimensions and orientation variation. (**b**) Optical leakage radiation microscopy setup. See the text for the details.

The randomized gratings have been designed as follows. We first created a set of 19 possible periods ($\Lambda _i \in [600 nm,1500nm]$) and used a computer program to scramble them in a random sequence. Rectangular apertures were distributed in a row with these distances. Then the relative rotation of the each aperture ($\Delta \theta _i$) was applied according to the condition of Eq. (2). Each row of apertures was repeated 19 times in the *y*-direction with a spacing of 1$\mu m$, thereby creating a non-random grating in that direction for reference. It was also hypothesized that two structures with opposite aperture rotation sense $\Omega$ would reverse the polarization dependence. To investigate this, for every right handed (RH) structure of counterclockwise aperture rotation we fabricated a left handed (LH) counterpart supporting clockwise rotation. Additionally, for each grating we designed a corresponding ordinary (O) hole array (where rectangular apertures were replaced by a circular hole while maintaining the period sequence) to serve as a reference structure. The actual gratings were milled in a gold film by focused ion beam (FIB) lithography in a 85 nm thick gold layer that was evaporated by sputtering on top of a 160 $\mu m$ glass cover slip. A total of 5 different randomized gratings triplets were fabricated in this way. A scanning electron micropscope (SEM) image of one such structure is shown in Fig. 2a. The dimensions of the rectangular aperture 150 nm $\times$ 250 nm were chosen to be sufficiently subwavelength yet anisotropic, as when the aspect ratio approaches zero (or infinity) either one of the dimensions must approach the near-wavelength regime, or the other must become too small to allow a significant amount of power through. The circular apertures were set to have the same area as their rectangular counterparts, requiring a radius of $\approx 100nm$. The images were captured by the leakage radiation microscopy (LRM) system^34,35^, depicted in Fig. 2b. The incident beam from the CW laser ($\lambda _0 = 785$nm at 20 mW), expanded by means of a telescope ($L_1$, $L_2$), was polarized by a linear polarizer (*LP*) followed by a quarter-wave plate (*QWP*) into a desired circular state. The objective ($O_{b,i}$, 20X, NA = 0.2) focused the laser beam onto the structure and an additional, oil immersion objective ($O_{b,o}$) was placed in contact with a substrate to extract the leakage radiation. The 100 mm tube lens (*TL*) was used to produce the real-space image, and a Fourier lens (*FL*) (50 mm) was added to image the *k*-space.

**Figure 3:**
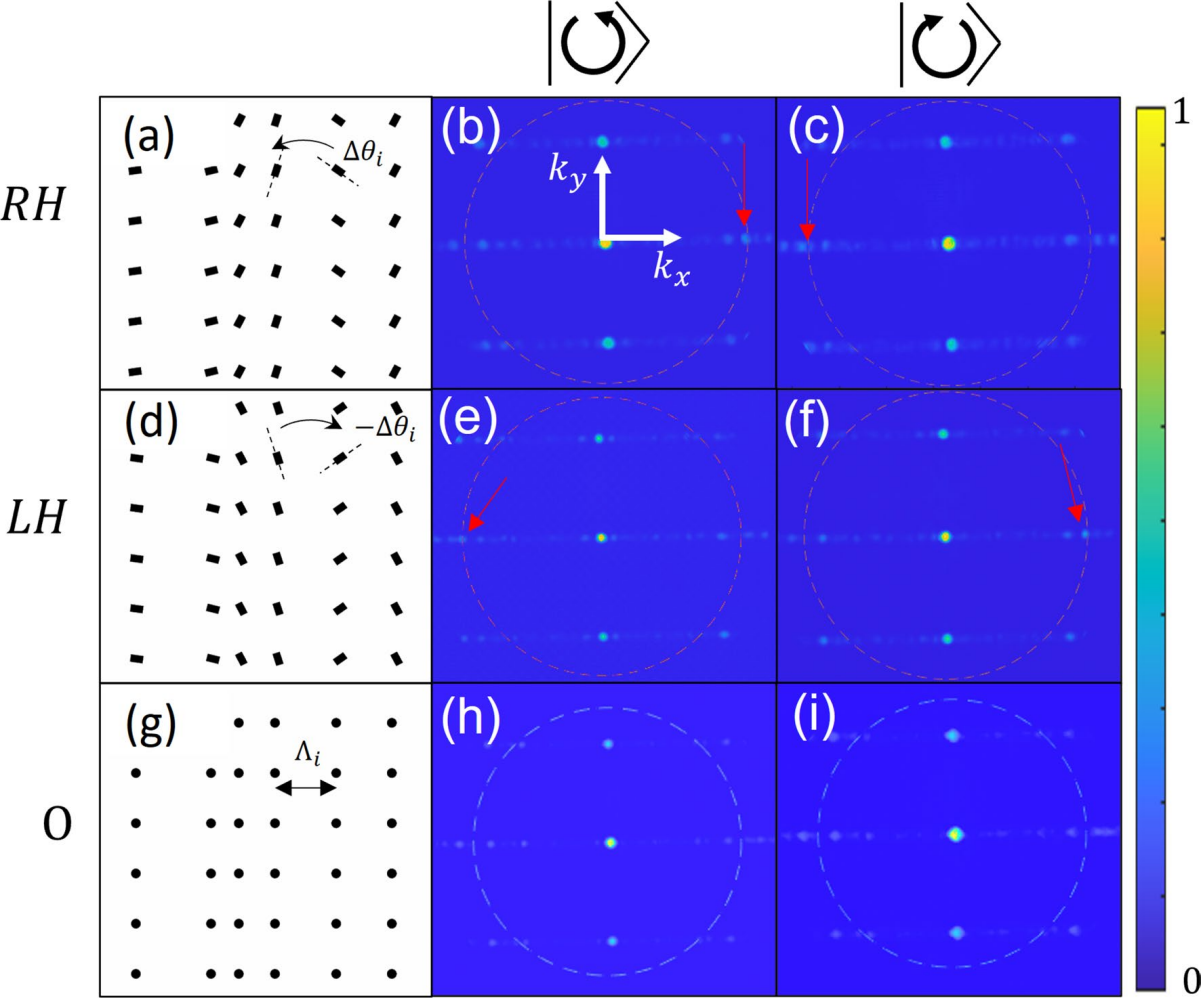
Typical measured *k*-space distributions for three types of the proposed structures. The structure geometries are shown in (**a**,**d**,**g**), (**b**,**e**,**h**) are the measured distributions for the incident RCP light and (**c**,**f**,**i**) show the corresponding measured intensities for the LCP light. Red arrows denote the location of the TD mode, when present. Gray dotted lines denote the plasmonic momentum $k_{SP}$. Colorbar on right denotes arbitrary intensity units.

Figure 3 shows *k*-space images of one out of the five structure triplets, comprising of the RH, LH and O structures illuminated with RCP and LCP, respectively. Hereafter we denote the RCP and LCP states as ${|{\circlearrowright }\rangle }$ and ${|{\circlearrowleft }\rangle }$. The dashed circle line depicts the location of the SP momentum $k_{SP}$ and panels (a,d,g) show the corresponding grating geometry. As can be seen, there are three clearly distinguishable lines spanning along the $k_x$ direction spaced according to the constant *y* periodicity. We note that there is no evident periodicity in the $k_x$ direction as would be expected when the Bloch theorem holds. While the light distribution is still somewhat discrete (which we relate to the finite nature of the random grating periods) one can, nevertheless, find this distribution rather uniform. By an accurate inspection of the spatially varying gratings we clearly recognize a polarization dependent intensity localization at $k_x = \pm k_{SP}$ (marked by the red arrows). Note, that for the RH structure, these modes lie at the $(\pm k_{SP},0)$ coordinate of the *k*-space for RCP, and LCP, respectively while for the LH structure, the modes flip their polarization dependence. For the non-rotating aperture structures, no significant localizations can be seen at these coordinates, and indeed no notable change is seen for different polarizations.

**Figure 4:**
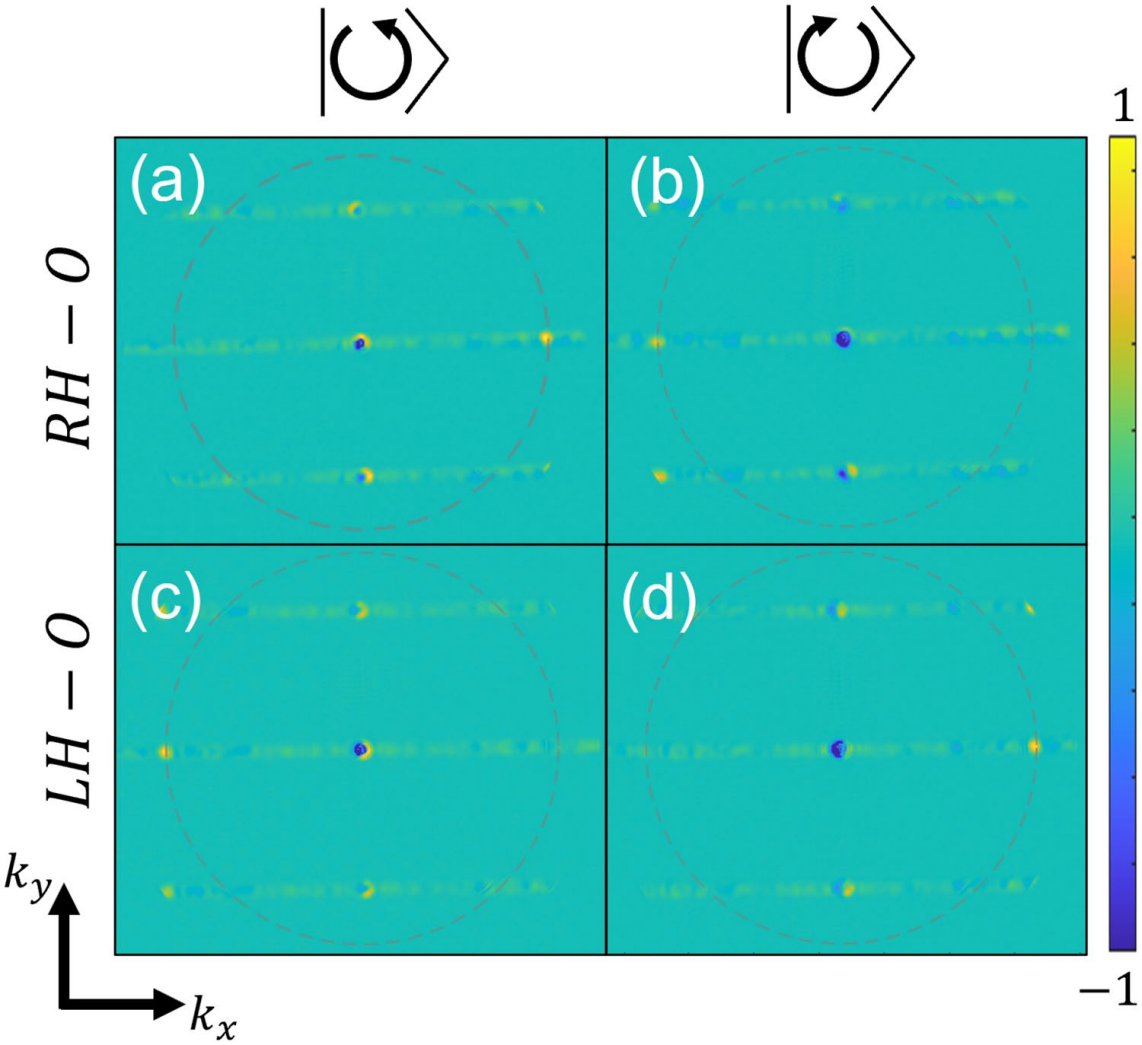
Averaged *k*-space distributions measured with different rotating gratings contrasted with the averaged results from the corresponding non-rotating structures. (**a**,**c**) Correspond to the RCP illumination and (**b**,**d**) for the LCP. Pictures (**a**,**b**) were taken from the right-handed structures and (**c**,**d**) were measured with the left-handed structures.

The finite ensemble of the randomized periods and the limited nature of the grating dimensions obviously reduce the effect of uniform smearing of OD in the *k*-space. In order to further improve the signal-to-noise ratio we average the distribution measured from the five non-rotating gratings and subtract it from the average of the five corresponding rotating gratings. We do this process separately for the RH and the LH structures and present the distributions in Fig. 4. As a result we are now able to clearly identify the topological plasmonic mode appearing at $k_x = \pm k_{SP}$ depending on the incident circular polarization. Also, it is evident that the opposite structure handedness leads to reversed distributions.

**Figure 5:**
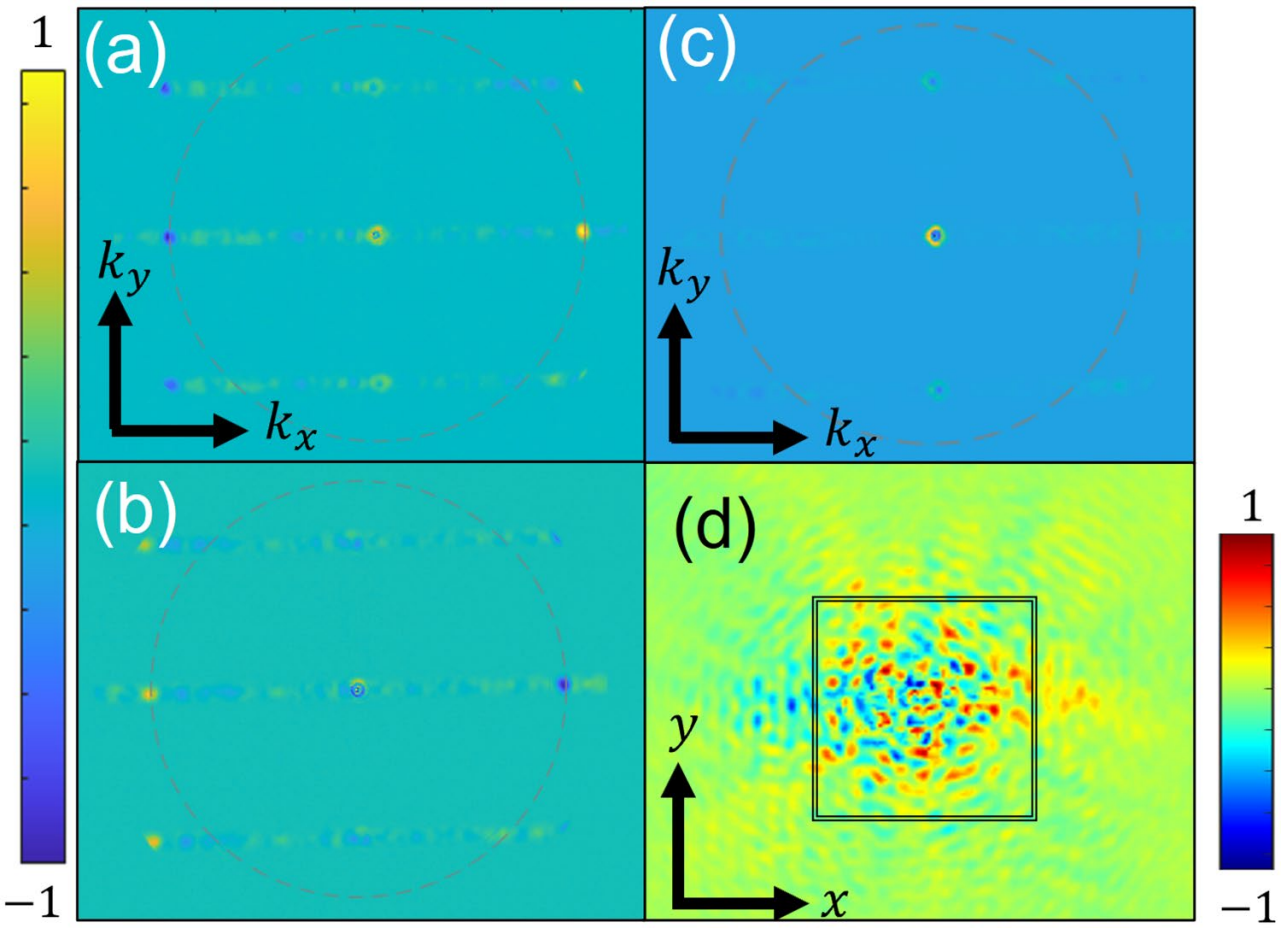
Measured averaged CD *k*-space maps for RH structure (**a**), LH structure (**b**) and the holey structure (**c**). The averaged real space image corresponding to (**a**) is shown in (**d**), with the black outline denoting the structure boundaries. Colorbar is shown in arbitrary units.

Finally, we demonstrate in Fig. 5 the polarization dependence of the structures in reference to themselves. Here we depict the local averaged circular dichroism map evaluated by $CD = \frac{\sqrt{I_{RCP}}-\sqrt{I_{LCP}}}{\sqrt{I_{RCP}}+\sqrt{I_{LCP}}}$ where $I_{RCP/LCP}$ stands for the *k*-space intensity distribution for RCP/LCP illumination. Figure 5a corresponds to the average CD of the five RH structures and Fig. 5b shows the average CD of the LH structures. For comparison, Fig. 5c presents the average measured CD map of the circular aperture structures where no polarization dependence is visible. We also studied the SP propagation through the grating in the real space (*x*, *y*). As the local combined phase imparted by the grating should lead to opposite propagation directions of the two circular states we expect to observe unequal distribution of intensity for illumination with RCP and LCP light. Indeed as can be seen in Fig. 5d on the left side of the grating (depicted by the frame) one can mostly find the LCP contribution while the right side is dominated by the RCP state. It should be noted that this result is the average of 5 distributions measured with different randomization sets, therefore clear plasmonic beam propagation was not expected in this experiment. Nevertheless, the polarization dependence of the measured real space perfectly corresponds to the distributions found above in the *k*-space.

## Conclusions

We have shown how local topological phases in randomized gratings can protect plasmonic modes. To test this, plasmonic structures comprised of randomly spaced and correctively rotating rectangular apertures were fabricated, along with corresponding circular reference structures. It was shown that randomizing the grating period severely reduces the Bloch effect, and that the combined dynamic and topological phases generated one, and only one pair of non-random, unidirectional, polarization dependent plasmons at $(\pm k_{SP},0)$ in the *x* direction. We have compared the behavior of the spatially-rotated gratings with reference structures comprising hole arrays with the same aperture spacing to ensure that the effect is topological in nature. The measured intensity distribution in the real space verified the unidirectional propagation of excited plasmonic waves through the grating structure. We believe that these structures can stand as a proof of concept for decoupling topological phases from other phases, opening the door to new applications and theoretical exploration of topological phenomena—both in photonics and in relevant analogues of solid state physics.

## Data Availability

All data generated or analyzed during this study are included in this published article.

## References

[1] Revah M, Yaroshevsky A, Gorodetski Y (2019). Spin-locking metasurface for surface plasmon routing. Sci. Rep..

[2] Huang L (2013). Helicity dependent directional surface plasmon polariton excitation using a metasurface with interfacial phase discontinuity. Light Sci. Appl..

[3] Jiang Q (2016). Directional and singular surface plasmon generation in chiral and achiral nanostructures demonstrated by leakage radiation microscopy. ACS Photonics.

[4] Jin D (2017). Infrared topological plasmons in graphene. Phys. Rev. Lett..

[5] Pietro PD (2013). Observation of Dirac plasmons in a topological insulator. Nat. Nanotech..

[6] Karch A (2011). Surface plasmons and topological insulators. Phys. Rev. B.

[7] You JW, Lan Z, Bao Q, Panoiu NC (2020). Valley-hall topological plasmons in a graphene nanohole plasmonic crystal waveguide. IEEE J. Sel. Top. Quantum Electron..

[8] Hofmann J, Sarma SD (2016). Surface plasmon polaritons in topological Weyl semimetals. Phys. Rev. B.

[9] Wang BX, Zhao CY (2020). Terahertz topological plasmon polaritons for robust temperature sensing. Phys. Rev. Mater..

[10] Appelbaum I, Drew HD, Fuhrer MS (2011). Proposal for a topological plasmon spin rectifier. Appl. Phys. Lett..

[11] Bliokh KY, Gorodetski Y, Kleiner V, Hasman E (2008). Coriolis effect in optics: Unified geometric phase and spin-hall effect. Phys. Rev. Lett..

[12] Bliokh KY, Rodríguez-Fortuño FJ, Nori F, Zayats AV (2015). Spin–orbit interactions of light. Nat. Photonics.

[13] Dahan N, Gorodetski Y, Frischwasser K, Kleiner V, Hasman E (2010). Geometric doppler effect: Spin-split dispersion of thermal radiation. Phys. Rev. Lett..

[14] Singh L, Fox M, Sternklar S, Gorodetski Y (2022). Topological diffraction from grating with space variant chirality. ACS Photonics.

[15] Maguid E (2018). Topologically controlled intracavity laser modes based on pancharatnam-berry phase. ACS Photonics.

[16] Epstein ED, Singh L, Fox M, Sternklar S, Gorodetski Y (2021). Topological dislocations for plasmonic mode localization in arrays of nanoscale rectangular au apertures: Implications for optical communications. ACS Appl. Nano Mater..

[17] Gorodetski Y, Drezet A, Genet C, Ebbesen TW (2013). Generating far-field orbital angular momenta from near-field optical chirality. Phys. Rev. Lett..

[18] Rajesh D, Nechayev S, Cheskis D, Sternklar S, Gorodetski Y (2018). Probing spin–orbit interaction via Fano interference. Appl. Phys. Lett..

[19] Berry MV (1984). Quantal phase factors accompanying adiabatic changes. Proc. R. Soc. Lond. Ser. A Math. Phys. Sci..

[20] Pancharatnam S (1956). Generalized theory of interference, and its applications. Proc. Ind. Acad. Sci. A.

[21] Bomzon Z, Kleiner V, Hasman E (2001). Pancharatnam-berry phase in space-variant polarization-state manipulations with subwavelength gratings. Opt. Lett..

[22] Fox M, Gorodetski Y (2022). Generalized approach to plasmonic phase modulation in topological bi-gratings. Appl. Phys. Lett..

[23] Agrawal VD, Lo YT (1972). Anomalies of dielectric-coated gratings. Appl. Opt..

[24] Heusinger, M., Banasch, M., Flügel-Paul, T. & Zeitner, U. D. Investigation and optimization of rowland ghosts in high efficiency spectrometer gratings fabricated by e-beam lithography. In *Advanced Fabrication Technologies for Micro/Nano Optics and Photonics IX*, vol. 9759, 22–28 (SPIE, 2016).

[25] Cumme M, Deparnay A (2015). From regular periodic micro-lens arrays to randomized continuous phase profiles. Adv. Opt. Technol..

[26] Morrison GR, Zhang F, Gianoncelli A, Robinson IK (2018). X-ray ptychography using randomized zone plates. Opt. Express.

[27] Stern, A., Rivenson, Y. & Javidi, B. Optically compressed image sensing using random aperture coding. In *Enabling Photonics Technologies for Defense, Security, and Aerospace Applications IV*, vol. 6975, 93–102 (SPIE, 2008).

[28] Biener G, Niv A, Kleiner V, Hasman E (2005). Geometrical phase image encryption obtained with space-variant subwavelength gratings. Opt. Lett..

[29] Lin A, Phillips J (2008). Optimization of random diffraction gratings in thin-film solar cells using genetic algorithms. Sol. Energy Mater. Sol. Cells.

[30] Trieu S, Jin X (2010). Study of top and bottom photonic gratings on GaN LED with error grating models. IEEE Quantum Electron..

[31] Draine BT, Flatau PJ (1994). Discrete-dipole approximation for scattering calculations. J. Opt. Soc. Am A.

[32] Angelsky, O. V., Magun, I. I., Maksimyak, P. P. & Perun, T. Spatial randomization of the scattered optical radiation. In *USSR-CSFR Joint Seminar on Nonlinear Optics in Control, Diagnostics, and Modeling of Biophysical Processes*, vol. 1402, 231–235 (SPIE, 1991).

[33] Angelsky OV, Maksimyak PP, Perun TO (1993). Dimensionality in optical fields and signals. Appl. Opt..

[34] Drezet A (2008). Leakage radiation microscopy of surface plasmon polaritons. Mater. Sci. Eng.: B.

[35] Drezet A (2007). Plasmonic crystal demultiplexer and multiports. Nano Lett..

